# Sympathetic Skin Response and Vasomotor Symptoms in Postmenopausal Osteoporotic Women

**Published:** 2015-07

**Authors:** Alireza Ashraf, Sharareh Roshanzamir, Ghahraman Bemana, Azam Mohammadi, Navid Jahani, Mahshid Naseri

**Affiliations:** 1 Shiraz Geriatric Research Center, Department of Physical Medicine and Rehabilitation, School of Medicine, Shiraz University of Medical Sciences, Shiraz, Iran;; 2 Department of Physical Medicine and Rehabilitation, Shiraz Burn Research Center, School of Medicine, Shiraz University of Medical Sciences, Shiraz, Iran

**Keywords:** Osteoporosis, Sympathetic nervous system, Autonomic dysfunction, Hot flashes

## Abstract

**Background:**

Osteoporosis is a common disease characterized by reduction in bone mass, due to depletion of calcium and bone protein. A pivotal role of the sympathetic nervous system in bone remodeling has been considered. On the other hand, elevated central sympathetic activation in postmenopausal women is involved in the creation of vasomotor symptoms. Also, sympathetic skin response (SSR) has been performed for evaluation of the peripheral and central autonomic nervous system dysfunctions. Therefore, to determine the association of the autonomic nervous system and osteoporosis, we evaluated the correlation between the bone mineral density (BMD) with the frequency of vasomotor symptoms and also sympathetic skin responses.

**Methods:**

This is a cross-sectional study in which thirty-three postmenopausal osteoporosis women, as the case group, and 31 age-matched postmenopausal women with normal BMD, as the control group, were included in our study. To evaluate the autonomic function, we assessed the frequency of vasomotor symptoms with a questionnaire and performed SSR test for the two groups. According to the parametrical or the nonparametrical distribution of the data, Independent Samples t-test or Mann Whitney test, respectively, were used to compare group differences.

**Results:**

The onset latencies of SSR recorded from both hands and feet were significantly prolonged in the case group compared with the control group (P<0.001). Amplitudes of SSR in the case group were significantly less than those of the control group (P<0.001). The postmenopausal osteoporotic women reported a significantly higher frequency of hot flashes and night sweats when compared with non-osteoporotic women (P<0.001).

**Conclusion:**

The higher frequency of vasomotor symptoms and impaired sympathetic skin responses in postmenopausal osteoporotic women suggests a role of autonomic dysfunction in osteoporosis.

## Introduction

Osteoporosis (OP) is a common disease, characterized by micro-architectural disruption and progressive loss of bone mass. The process of the bone remodeling is controlled by hormonal factors, nutritional status and biomechanical stress.^1-3^ Systemic hormones and local factors regulate osteoblastic and osteoclastic activities.^3^ Leptin, a protein hormone, is released by adipocytes and regulates bone metabolism. It has been demonstrated that leptin binds to hypothalamic receptor and not only regulates the osteoblastic and osteoclastic activities, but also controls the release of noradrenalin by the sympathetic nervous system (SNS).^3^^,^^4^

It has been shown that there is a close relationship between the sympathetic nervous system and bone metabolism. Excessive sympathetic nervous activity results in bone loss via increased bone resorption and decreased bone formation.^2^^,^^5^^,^^6^

Several studies have revealed the expression of β2-adrenergic and β1-adrenergic receptors in human periosteal osteoblasts and osteoclast-like cells.^7^ Furthermore, sensory and sympathetic nerve fibers supply the bone and periosteum.^3^

Several investigators suggest that low dose β-adrenergic blocking agents can improve the bone loss and bone fragility.^2^^,^^3^^,^^7^ It confirms the role of sympathetic nervous system in the bone remodeling.

On the other hand, several studies reported that women with vasomotor symptoms have greater levels of brain norepinephrine (NE) when compared with asymptomatic women.^8^Experimental animal research indicated that greater levels of the brain NE are related to a narrowing of the thermoneutral zone.^9^ It has been theorized that estrogen withdrawal in menopause may influence adrenergic receptors.^10^ Menopausal women report the vasomotor symptoms most commonly. These symptoms, so called hot flashes, include a sudden increase of blood flow to the face, neck, and chest, the sensation of extreme heat and profuse sweating. When symptoms occur at night, they are referred as “night sweats” and can cause significant sleep disturbances. Episodes of vasomotor symptoms can last 1 to 5 minutes and can be associated with perspiration, flushing, chills, anxiety, and even heart palpitations. The vasomotor symptoms in postmenopausal women, the measure of autonomic dysfunction, were associated with reduced bone mass.^11^

The sympathetic skin response is being used to assess the autonomic dysfunction. SSR, one of the most frequently used measures in neurophysiological studies, is a slow wave and reliable test recording of sympathetic efferent fibers stimulation.^12^ SSR has been evaluated in the peripheral and central autonomic nervous system dysfunctions, such as spinal cord injury and stroke.^13^^,^^14^ Liguori et al. in 2011 investigated the ability of microneurography and the corresponding skin organ effector responses (sympathetic skin response and skin vasomotor reflex) to diagnose autonomic dysfunction in patients with selective small nerve fiber involvement of different origin with and without autonomic symptoms. They demonstrated that SSR and skin vasomotor reflex (SVR) can reveal autonomic involvement in small fiber neuropathy with a good sensitivity.^15^

Tosun et al. in 2011 studied SSR and heart rate variability (HRV), evaluating the presence of autonomic dysfunction in postmenopausal osteoporotic women and found significant prolonged latencies of SSR recorded from both hands and decreased SSR amplitudes of the right hand and both feet, and differences in 24-hr frequency domain measure as a parasympathetic activity marker in osteoporotic women compared with non-osteoporotic women. They suggested that autonomic dysfunction may be present in osteoporosis.^16^ Therefore, to confirm the relationship between the autonomic dysfunction and osteoporosis, we evaluated the correlation between bone mineral density (BMD) with the frequency of vasomotor symptoms, another aspect of autonomic dysfunction, and also sympathetic skin responses in postmenopausal women.

## Materials and Methods

*Methods*

This cross-sectional study was performed during January 2013 and November 2013. Based on the data of similar studies, power=90%, α=0.05 and confidence interval=95%, the sample size was calculated as 25 in each group. We selected 50- 65 year old women who referred to physical medicine and rehabilitation outpatient clinics of Shahid Faghihi Hospital through simple sampling method if everyone had experienced menopause for at least one, but not more than 10 years. The patients were excluded with the history or diagnosis of systemic diseases (obesity (BMI≥30 Kg/m^2^), diabetes mellitus, thyroid dysfunction,  malignancy, hypertension), secondary osteoporosis, prior hormone therapy, usage of drugs (including calcium-channel blockers, beta-blockers, lipid-lowering agents, glucocorticoids, anti-diabetic agents, antiresorption agents, anti-arrhythmia agents, oral contraceptives, anticoagulants, diuretics), neurological and psychiatric diseases, diagnosis of peripheral neuropathy or other neuropathies in nerve conduction study, chemotherapy or radiotherapy, and usage of alcohol and tobacco.

This study was approved by the Ethics Committee of our university and all the participants who were included in this study gave written informed consent to take part in the survey. The medical history of patients was taken before enrollment and all women underwent a physical examination for evaluation of exclusion criteria, and then we tested them for laboratory investigations, including complete blood count, erythrocyte sedimentation rate, C-reactive protein, calcium, phosphorus, alkaline phosphatase, rheumatoid factor, FBS, PTH, FSH, thyroid function tests, and vitamin D level.

Dual-energy x-ray absorptiometry (DEXA) measurement was used to diagnose osteoporosis in patients. Measurement of BMD for two groups was performed using the same dual-energy x-ray absorptiometer (the Lunar DPX-L model, Lunar, Madison, Wisconsin, USA). Areal BMD is described in absolute terms of grams of mineral per square centimeter scanned (g/cm2) and as a relationship to two norms: Z-score, the expected BMD for the patient’s age and sex or T-score, compared to young healthy adults of the same sex. The difference between the patient’s score and the norm is described in standard deviations (SD) above or below the mean. A decline in BMD expressed in absolute terms (g/cm2) or in standard deviations (T-scores or Z-scores) begins during young adulthood, advances in women at menopause and postmenopausal women and men aged 50 and older. Based on BMD measurement at the spine, hip or forearm by DEXA devices, the World Health Organization defines normal as BMD within 1 SD of a “young normal” adult (T-score at -1.0 and above), osteopenia as BMD between 1.0 and 2.5 SD below that of a “young normal” adult (T-score between -1.0 and -2.5), osteoporosis as BMD 2.5 SD or more below that of a “young normal” adult (T-score at or below -2.5) and severe or established osteoporosis as the patients have already experienced one or more fractures.^17^ Thirty-three postmenopausal women with osteoporosis as case group and 31 postmenopausal women with normal BMD as control group were enrolled in this study.

We assessed the frequency of vasomotor symptoms with a questionnaire in the case and control groups, including the number of days that a patient had hot flashes in the last week, the average number of hot flashes per day, the highest amount of hot flashes per day, the number of nights that a patient had nocturnal awakening in the last week because of night sweats, and the number of waking up per night because of night sweats.^11^

To measure SSR, we performed the tests by using a Medelec Synergy electromyography instrument (VIASYS Healthcare UK, manor way, old woking, surrey, UK) and surface disc electrode for recordings in a silent room at a temperature between 22˚C and 24˚C in which the patients lied in supine and resting position. The skin temperature during all the tests was 32˚C. First, we did nerve conduction studies to exclude peripheral neuropathies; then, the active and reference electrodes were secured to the palm and the back of the hand and the sole and dorsum of the foot according to the standard method.^18^ To avoid habituation, the stimuli were delivered at irregular intervals over one minute. The measured latency and amplitude were defined as the onset of the first negative deflection and the base-to-peak value of the recorded waves, respectively. A band pass of 0.1-1 KHz, a sweep speed of 500 ms/div and a sensitivity of 200 μV/div were used.

*Statistical Analysis*

We analyzed the obtained data by using the statistical package for the social sciences (SPSS), version 16. Data were reported as mean±SD. According to the parametrical or the nonparametrical distribution of the data, Independent Samples t-test or Mann Whitney test, respectively, were used to compare group differences. P≤0.05 was considered statistically significant. 

## Results

There was no statistically significant difference in demographic characteristics including age, body mass index, duration of menopause and parity between the two groups. (Table 1) Laboratory studies were within normal ranges.  The mean ages of the case and control groups were 55.7±4.4 (rang, 52–63 years) and 54±4.2 years (rang, 50–63 years), respectively. (P>0.05) Bone mineral densities of the cases were significantly decreased compared with the control group (Table 2).

**Table 1:** Demographic characteristics of the case and control groups

	**Case group(n=33)**	**Control group (n=31)**	P value^*^
b	55.757±4.458	54.064±4.234	0.125
BMI, Kg/m^ 2 ^	28.648±2.896	29.632±2.9278	0.182
Duration of menopause, yr	4.984±2.466	4.516±2.557	0.458
Number of gravity	3.393±1.853	4.064±1.860	0.154

^*^t test, significant level set as P<0.05; BMI: Body mass index.

**Table 2:** Bone Mineral Density in the case and control groups

	**Case group(n=33)**	**Control group (n=31)**	P value^*^
Femoral neck, g/cm^2^	0.744±0.076	0.985±0.086	<0.001
A-P L2-4 lumbar vertebrae, g/cm^ 2 ^	0.7560±0.086	1.026±0.036	<0.001

^*^t test, significant level set as P<0.05; A-P: Anteroposterior

96.9% and 90.9% of the cases and 77.4% and 41.9% of the controls reported hot flashes and night sweats, respectively. Analysis of data indicated a statistically significant difference between the two groups in the frequency of hot flashes and night sweats. (Table 3) The postmenopausal osteoporotic women reported a significantly higher frequency of hot flashes and night sweats when compared with non-osteoporotic women.

**Table 3:** Frequency of hot flashes and night sweats in the case and control groups

	**Case group (n=33)**	**Control group (n=31)**	** P value^a^**
Number of hot flashes per 24 hours	2.878±1.317	1.322±0.979	<0.001
Number of hot flashes in the last week	4.212±1.781	2.193±1.492	<0.001
Number of night sweats per night	1.212±0.649	0.483±0.625	<0.001^b^

^a^t test, significant level set as P<0.05; ^b^Mann-Whitney test is used for analysis of number of night sweats, significance level set at P<0.05

The mean onset latencies of SSR recorded from both hands and feet were significantly prolonged in the case group compared with the control group (P<0.001). Decreased amplitudes of SSR in the case group were statistically significant compared with the control group (P<0.001) (Table 4).

**Table 4:** Sympathetic skin responses data of the upper and lower limbs in the case and control groups

	**Case group (n=33)**	**Control group (n=31)**	P value^*^
Onset latency (second)			
Right hand	1.570±0.135	1.429±0.131	<0.001
Left hand	1.575±0.124	1.431±0.108	<0.001
Right foot	2.009±0.207	1.772±0.183	<0.001
Left foot	1.976±0.204	1.754±0.148	<0.001
Amplitude (µv)			
Right hand	684.1±140.3	932±157	<0.001
Left hand	701.8±134	934.1±133.6	<0.001
Right foot	483.2±106.1	641.3±201.1	<0.001
Left foot	491±122.4	659.4±187.1	<0.001

^*^t test, significance level set at P<0.05

There was no statistically significant correlation between the frequency of vasomotor symptoms and reduction value of bone mineral density within the case group. 

## Discussion

The frequency of vasomotor symptoms was associated with low BMD. The postmenopausal osteoporotic women reported a significant higher frequency of hot flashes and night sweats when compared with non-osteoporotic women.

The onset latencies of SSR recorded from both hands and feet were significantly prolonged in the case group compared with the control group. Amplitudes of SSR in the case group were significantly less than those of the control group.

The adrenergic and cholinergic activity of the sympathetic nervous system has been shown to be negative and positive regulators of bone mass, respectively. Adrenergic signaling suppresses the osteoblast reproduction and advances osteoclastogenesis.^19^^,^^20^ Pierroz et al. concluded that an adrenergic antagonist might prevent bone loss in ovariectomized mice and revealed synergistic effects with PTH-induced bone formation.^21^ Bonnet et al. evaluated the preventive effect of propranolol on the bone in ovariectomized rats and reported that the beta blockers were correlated with a higher BMD at the hip and lumbar vertebrae.^22^ The existence of adrenoreceptors in the bone indicates that the sympathetic nervous system might play a role in bone metabolism.^23^

A diminution in suppression of presynaptic *α*_2-_receptors leads to raised brain NE levels, which leads to a high frequency of hot flashes.^24^ Gast et al. demonstrated that vasomotor symptoms are associated with decreased bone density.^11^ These findings show that sympathetic activation is higher in women with vasomotor and there is a link between hot flashes and a reduced bone mass. Prolonged latencies of SSR recorded from both hands and feet in the case group supported the presence of an autonomic dysfunction in osteoporosis.

This study evaluated the autonomic dysfunction via assessment of frequent vasomotor symptoms and SSR in postmenopausal osteoporotic women. We found a similar study that assessed heart rate variability (HRV) and SSR in osteoporosis in English literature. Tosun et al. examined HRV and SSR to evaluate autonomic function in postmenopausal osteoporotic women and postmenopausal non-osteoporotic women. They reported significantly increased SSR latencies of both hands, decreased SSR amplitudes of the right hands and both feet, a decreased 24-hr high-frequency value, and an increased 24-hr low-frequency/high-frequency (LF/HF) in the postmenopausal osteoporotic women compared with postmenopausal non-osteoporotic women.^16^ In our study, the number of cases and controls was more than the previous study. However, to determine the pathophysiologic mechanisms, diagnostic and therapeutic significance of autonomic dysfunction in osteoporosis, new studies are needed. The limitations of our study are lack of assessment of the serum norepinephrine, leptin, 24-hour urine calcium and estrogen levels; we also did not examine the association between vasomotor symptoms and SSR with BMD in osteopenic patients and we had better choose SSR test with other scales to evaluate autonomic dysfunction. The onset of menopause in our participants was between 1-10 years before this study, and it was difficult to remember the frequency of vasomotor symptoms in perimenopausal period, so to avoid recall bias we evaluated these symptoms in the week before doing the SSR test. Further prospective research is needed to evaluate vasomotor symptoms, HRV and SSR associated with BMD that begins at perimenopausal period.

## Conclusion

In conclusion, our results showed the association of impaired sympathetic skin response (prolonged onset latencies) and higher frequency of vasomotor symptoms with low BMD. Identifying vasomotor symptoms and impaired SSR test in perimenopausal and postmenopausal period may be suggested as findings for BMD evaluation. Sympathetic nervous system evaluation can help to identify new therapies and may be able to prevent or improve bone loss. 
